# Analysis of Structures of SARS-CoV-2 Papain-like Protease Bound with Ligands Unveils Structural Features for Inhibiting the Enzyme

**DOI:** 10.3390/molecules30030491

**Published:** 2025-01-23

**Authors:** Ann Varghese, Jie Liu, Bailang Liu, Wenjing Guo, Fan Dong, Tucker A. Patterson, Huixiao Hong

**Affiliations:** National Center for Toxicological Research, U.S. Food and Drug Administration, Jefferson, AR 72079, USA; ann.varghese@fda.hhs.gov (A.V.); jie.liu1@fda.hhs.gov (J.L.); bailang.liu@fda.hhs.gov (B.L.); wenjing.guo@fda.hhs.gov (W.G.); fan.dong@fda.hhs.gov (F.D.); tucker.patterson@fda.hhs.gov (T.A.P.)

**Keywords:** SARS CoV-2, papain-like protease, PLpro, ligands, binding sites, 3D structures

## Abstract

The COVID-19 pandemic, driven by the novel coronavirus SARS-CoV-2, has drastically reshaped global health and socioeconomic landscapes. The papain-like protease (PLpro) plays a critical role in viral polyprotein cleavage and immune evasion, making it a prime target for therapeutic intervention. Numerous compounds have been identified as inhibitors of SARS-CoV-2 PLpro, with many characterized through crystallographic studies. To date, over 70 three-dimensional (3D) structures of PLpro complexed ligands have been deposited in the Protein Data Bank, offering valuable insight into ligand-binding features that could aid the discovery and development of effective COVID-19 treatments targeting PLpro. In this study, we reviewed and analyzed these 3D structures, focusing on the key residues involved in ligand interactions. Our analysis revealed that most inhibitors bind to PLpro’s substrate recognition sites S3/S4 and SUb2. While these sites are highly attractive and have been extensively explored, other potential binding regions, such as SUb1 and the Zn(II) domain, are less explored and may hold untapped potential for future COVID-19 drug discovery and development. Our structural analysis provides insights into the molecular features of PLpro that could accelerate the development of novel therapeutics targeting this essential viral enzyme.

## 1. Introduction

The coronavirus disease 2019 (COVID-19) pandemic, caused by the novel coronavirus severe acute respiratory syndrome coronavirus-2 (SARS-CoV-2), significantly altered the global socioeconomic landscape [1,2]. Originating in the city of Wuhan, China [3,4], the virus rapidly spread worldwide, resulting in over 776 million reported COVID-19 cases as of 11 October 2024, according to the World Health Organization (https://data.who.int/dashboards/covid19/cases, accessed on 1 November 2024). SARS-CoV-2 primarily transmits via respiratory droplets or close interpersonal contact, with a predilection for infecting the respiratory tract [3]. During the pandemic, rapid viral replication led to the development of variants and subvariants, many with increased transmissibility and potential immune escape. Noteworthy variants of concern include the Alpha, Beta, Gamma, Delta, and Omicron [4,5,6,7,8,9,10].

Despite its high transmissibility, SARS-CoV-2 exhibits a relatively low mortality rate (1.2% for the week ending 2 November 2024 in USA: covid.cdc.gov/covid-data-tracker/, accessed on 15 November 2024) compared to other members of the coronaviridae family, such as SARS-CoV from the 2002 outbreak which had a mortality rate of 10.87%, and the middle east respiratory syndrome coronavirus (MERS-CoV) from the 2012 outbreak with a mortality rate of 34.77% [11,12,13]. Although SARS-CoV-2 is less lethal than these viruses, its high transmissibility posed public health challenges. Today, the global threat posed by COVID-19 has lessened, largely due to the broad distribution of vaccines. However, the continuous emergence of vaccine-resistant variants highlights the enduring risk and underscores the importance of developing effective treatments.

At the molecular level, SARS-CoV-2 shares approximately 79% of its genome identity with SARS-CoV and 50% with MERS-CoV [14,15]. It is a positive-sensed single-stranded RNA virus composed of approximately 30,000 nucleotides [16,17]. The genome contains fourteen functional open reading frames (ORFs) that encode structural, nonstructural, and accessory proteins. At the 3′ end of the genome, four structural and eight accessory proteins are located, while the 5′ end comprises nonstructural proteins (nsps). Two large ORFs, ORF1a and ORF1b, cover two-thirds of the genome, encoding sixteen nsps critical to viral infectivity and pathogenicity [18,19].

SARS-CoVs rely on the structural spike protein, located on the viral surface, to initiate infection [20,21,22]. The spike protein mediates viral entry into host cells by interacting with the angiotensin-converting enzyme 2 (ACE2) receptor [23,24,25,26,27]. Once inside the host, viral maturation, replication, and invasion are facilitated by two key functional cysteine proteases: the 3-chymotrypsin-like main protease and papain-like protease (PLpro) [28,29,30]. The main protease is released from nsp5 via autocleavage and catalyzes polyprotein hydrolysis at 11 sites (nsp4-nsp16), while PLpro, released from nsp3, hydrolyzes peptide bonds between nsp1/nsp2, nsp2/nsp3, and nsp3/nsp4, releasing individual functional nsps critical for various stages of the viral life cycle [31,32,33,34,35].

PLpro is located between the SARS unique domain (SUD) and the nucleic acid binding domain (NUB) of nsp3, one of the largest nonstructural proteins with a molecular weight of approximately 212 kDa and consisting of 1945 amino acid residues [36,37]. PLpro exhibits a preference for cleaving peptide bonds following the P4-P1 Leu-X-Gly-Gly sequence motif (where X = Lys, Arg, or Asn) [34,38,39]. This cleavage site is highly conserved among SARS-CoV-2, SARS-CoV, and MERS-CoV [34]. In addition to its role in viral polypeptide cleavage, SARS-CoV-2 PLpro exhibits two other proteolytic activities: removal of ubiquitin (Ub) and ubiquitin-like interferon-stimulated gene 15 (ISG15) modification from host proteins [40,41]. Ub is a conserved regulatory protein in eukaryotes [42], and ISG15 is a Ub-like protein modifier with two Ub-like domains connected via a peptide. Both Ub and ISG15 have an LXGG sequence fragment at their C-terminus, recognized and cleaved by PLpro. By removing Ub and ISG15 from host proteins, PLpro suppresses antiviral signaling pathways, weakening the host’s defense mechanisms against SARS-CoV-2 infections. This dual role as a deubiquitinase and deISGylase highlights PLpro’s interference with innate immune responses, making it a prime target for drug discovery [43,44,45,46]. Targeting proteases involved in peptide cleavage has proven successful in treating viral infections caused by human immunodeficiency virus [47] and hepatitis C virus [48].

Despite its significance, SARS-CoV-2 PLpro has not been fully leveraged in drug discovery. Currently, only two antiviral drugs have been approved for treating COVID-19: Veklury^®^ (remdesivir), which inhibits RNA-dependent RNA polymerase (RdRp), and Paxlovid™ (nirmatrelvir and ritonavir), which targets the main protease [49,50,51]. Lagevrio™ (molnupiravir), another RdRp inhibitor, is under emergency use authorization and has not yet received full FDA approval [52]. Notably, no antiviral drugs specifically targeting PLpro have been approved. Therefore, there is an urgent need to discover compounds that target PLpro with favorable pharmacokinetic properties.

Given the lack of approved PLpro inhibitors and the urgent need for rapid solutions, drug repurposing presents an efficient strategy. It offers a fast and cost-effective approach compared to the conventional process of drug discovery [53,54,55]. Many therapeutic agents reported for SARS-CoV-2 PLpro are repurposed molecules. One of the earliest and widely studied non-covalent inhibitors of SARS-CoV-2 PLpro is 5-amino-2-methyl-N[(1R)-1-naphthalen-1-ylethyl]benzamide (GRL0617) [56,57]. Initially effective against SARS-CoV PLpro, its efficacy was extended to SARS-CoV-2 PLpro due to the structural and biological similarities between the two proteases [56,58]. Computational techniques like molecular docking and bioinformatics have identified drugs with strong binding affinity to SARS-CoV-2 PLpro [59,60,61], including various antibacterial, antiviral, and asthmatic drugs. Compounds containing aldehydes, ketones, epoxy ketones, activated esters, and similar head groups have shown effectiveness in inhibiting SARS-CoV PLpro and are being explored for their potential to inhibit SARS-CoV-2 PLpro [34].

Computational chemistry techniques such as molecular docking [62,63,64,65] and molecular dynamics simulations [66,67,68,69,70] play a pivotal role in drug discovery and development. To advance drug repurposing efforts and guide the development of new PLpro inhibitors through computational chemistry, a deeper understanding of the enzyme’s structure and its binding pockets is essential. These pockets where inhibitors bind must meet specific structural requirements. Identifying and characterizing their shape, size, and residue composition is crucial for determining how inhibitors can interact with the enzyme. This information is often obtained through techniques such as X-ray crystallography [71], NMR spectroscopy [72], or homology modeling [73]. To date, over 70 three-dimensional (3D) structures of SARS-CoV-2 PLpro, including wild-type proteins, mutants, enzyme–substrate complexes, and enzyme–inhibitor complexes, have been resolved and are available in the Protein Data Bank (PDB). All these structures have been resolved exclusively through X-ray crystallography; no entries derived from other methods have been reported. This rich repository of 3D structural data offers ample opportunities for investigating the structural characteristics and binding specificities of PLpro.

In this article, we present a comprehensive analysis of the 3D structures of SARS-CoV-2 PLpro, with a particular focus on its binding sites.

## 2. Structural Basis of SARS-CoV-2 PLpro Enzymatic Function

**Figure 1:**
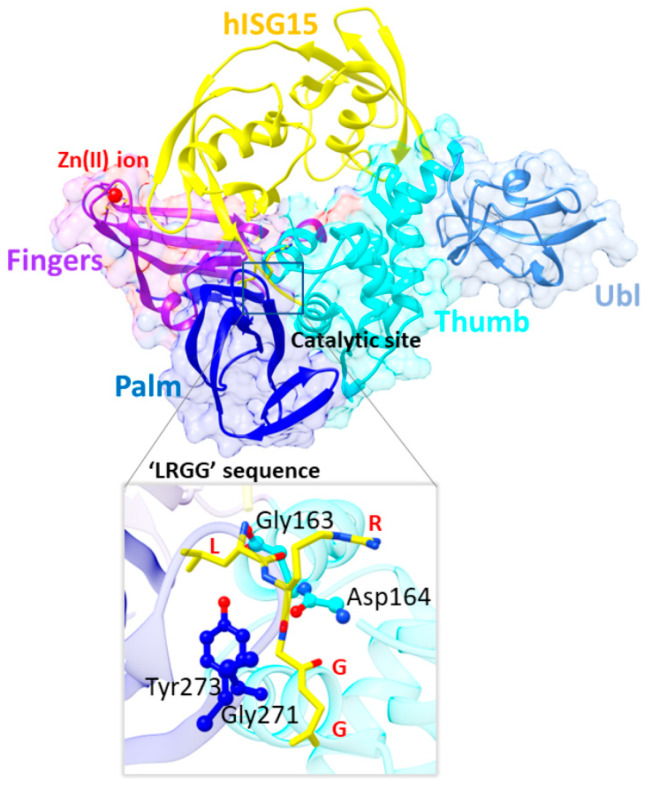
Structure of SARS-CoV-2 PLpro complexed with human ISG15 (PDB ID: 7RBS). The domains are color-coded for clarity: Ubl (sky blue), thumb (cyan), fingers (violet), Zinc ion (red ball), palm (blue), and ISG15 (yellow). The enlarged view highlights the key residues within the protein that interact with the ‘LRGG’ peptide of the substrate. The protein residues are shown in a ball-and-stick model, while the substrate ‘LRGG’ tetrapeptide is represented in a stick model.

SARS-CoV-2 PLpro consists of residues 746–1060 from nsp3, the largest nsp encoded by the virus [39]. Structurally, the PLpro monomer contains two main domains: the ubiquitin-like (Ubl) domain and the catalytic domain, which adopts a right-handed “thumb–palm–fingers” architecture (Figure 1). This arrangement is common to ubiquitin-specific proteases despite their low sequence similarity (approximate 10%) to PLpro [74,75].

**Figure 2:**
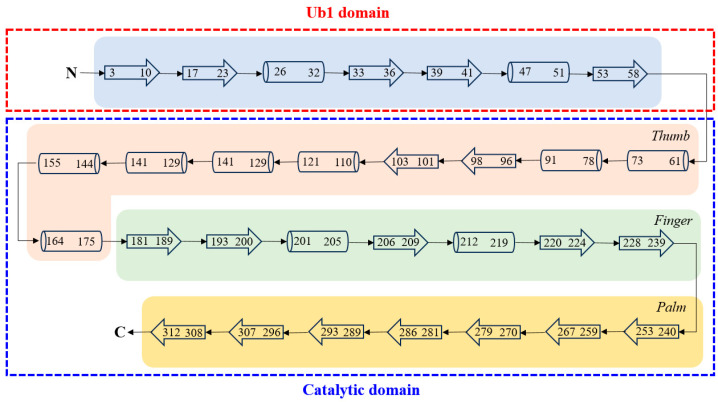
The two-dimensional structure of SARS CoV-2 PLpro. The Ubl domain is indicated by the red rectangle, while the catalytic domain is included in the blue rectangle. The three subdomains of the catalytic domain are marked in different colors. The cylinders represent α helices, and the larger empty arrows indicate β strands.

The Ubl domain, spanning 60 residues, includes five β strands and two α helices. While the exact function of this domain remains unclear, research has identified certain residues that are involved in substrate binding, particularly with ISG15 or K48-linked diubiquitin [40]. The Ubl domain is connected to the catalytic domain of SARS CoV-2 PLpro (Figure 2, generated according to PDBsum [76], https://www.ebi.ac.uk/thornton-srv/databases/cgi-bin/pdbsum/GetPage.pl, accessed on 13 January 2025).

The larger catalytic domain is further divided into three subdomains—thumb, palm, and fingers (3D structure shown in Figure 1 and 2D structure given in Figure 2). The thumb subdomain (residues 61–175) is composed of seven α helices and two β hairpins, while the palm subdomain (residues 241–315) consists of seven β strands. The catalytic site, located at the interface of the thumb and palm subdomains, features a catalytic triad composed of Cys111, His272, and Asp286, and is responsible for peptide bond hydrolysis [34,74]. A hydrogen bond between His272 and Asp286 helps orient His272 for catalysis. Additional interactions in the active site include hydrogen bonds between Cys111 and Thr115, His272 and Thr265, and Asp286 and Leu289. Notably, SARS-CoV-2 PLpro shares 90% active site sequence similarity with SARS-CoV PLpro [39,77]. The finger subdomain (residues 181–239) is composed of five β strands and two α helices and houses a Zn(II) site, coordinated by four cysteine residues (Cys189, Cys192, Cys224, and Cys226). This Zn(II) site is crucial for maintaining the structural integrity of PLpro and is essential for its enzymatic function.

## 3. Substrate Specificity and Catalytic Cycle

The primary substrates targeted by SARS-CoV-2 PLpro are the peptides between nsp1/nsp2, nsp2/nsp3, and nsp3/nsp4, all sharing the conserved Leu-Asn-Gly-Gly motif [78]. In this tetrapeptide sequence, leucine occupies the P4 position, while glycine is found at P1, with P4–P1 representing the residues preceding the cleavage site. Hydrolysis specifically targets the P1–P1′ bond, where the P1′ residue is typically alanine in nsp1/nsp2 and nsp2/nsp3, and lysine in nsp3/nsp4.

**Figure 3:**
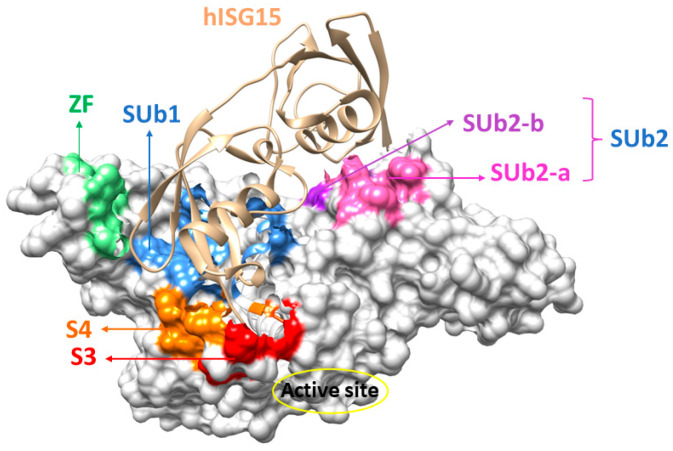
Common ligand binding sites in SARS-CoV-2 PLpro: S3 (red), S4 (orange), SUb1 (blue), SUb2-a (pink), SUb2-b (violet), and Zn(II) fingers (ZFs) (parrot green). The secondary substrate structure of the human ISG15 is shown in brown.

PLpro exhibits a high degree of substrate specificity, particularly at the P1 and P2 positions, exclusively accommodating glycine residues that bind to the enzyme’s S1 and S2 sites. The presence of two glycine residues creates a shallow and confined region that limits access to the active site. In contrast, the P3 position is more flexible, occupying the S3 binding site (Figure 3) which can accommodate both hydrophobic and positively charged residues. At the P4 position, only hydrophobic residues such as lysine are allowed [79] with P4 binding to the S4 site (Figure 3), which is relatively flexible and large allowing for long side-chain amino acids. Together, the S1–S4 sites represent the four primary substrate binding regions involved in recognizing the peptide sequence.

In addition to cleaving the nsps, SARS-CoV-2 PLpro also processes Ub and ISG15. The SUb1 site (Figure 3), enclosed by the fingers, palm, and thumb subdomains, primarily recognizes the monoubiquitin substrate and the C-terminal domain of ISG15. Meanwhile, the SUb2 (Figure 3), located near the conserved S2 helix (residues 60–70) within the thumb domain but adjacent to the Ubl domain, binds polyubiquitin and the N-terminal domain of ISG15. Interestingly, PLpro shows a higher affinity for ISG15 substrates over Ub [74,80]. Both Ub and ISG15 feature a conserved ‘LXGG’ recognition motif at their C-terminus, which extends into the active site. At the variable P3 position within this motif, an arginine residue is typically present. During the cleavage of the P1-P1′ bond in the Ub/ISG15 substrates, the P1′ residue usually belongs to the host protein, except in the K48-linked polyUb and ISG-15 modified substrates, where the cleavage occurs between the C-terminal and a lysine residue of the substrate [80].

**Figure 4:**
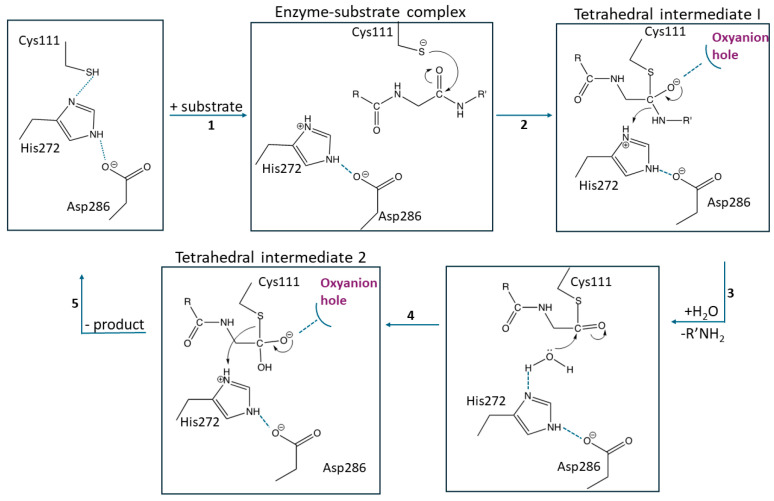
General catalytic mechanism and intermediates of SARS CoV-2 PLpro.

The catalytic cycle of SARS-CoV-2 PLpro follows a cysteine protease mechanism, with Cys111 serving as a nucleophile, His272 acting as a general acid-base, and Asp286 aiding in orienting His272 to facilitate the deprotonation of Cys111. In the initial step of proteolysis, the thiol group of Cys111 is deprotonated by the imidazole group of His272 [34]. While no experimental data exist on the protonation state of Cys111 before the nucleophilic attack, the deprotonated Cys111 attacks the electrophilic carbonyl of the scissile peptide bond forming an oxyanion tetrahedral intermediate (Figure 4). This intermediate is stabilized by an adjacent oxyanion hole within the active site, with Trp106 likely playing a key role in its stabilization, akin to the role of Trp107 in SARS-CoV PLpro [35,40,81]. The oxyanion hole is vital for stabilizing the corresponding transition state, ensuring efficient catalysis. Subsequently, His272 transfers a proton to the C-terminal amine of the substrate, resulting in the cleavage of the peptide bond. A water molecule then attacks the carbonyl carbon of the thioester intermediate, forming a second tetrahedral intermediate. Finally, Cys111 dissociates, releasing the carboxylic acid fragment of the substrate and restoring the enzyme to its original state [82].

## 4. Structures of SARS CoV-2 PLpro

There are 71 3D structures of SARS-CoV-2 PLpro, determined by X-ray crystallography, in the PDB. Of these, four structures correspond to the wild-type enzyme in its apo form (PDB IDs: 6W9C, 6WZU [74], 7NFV [83], and 8FWO), while eight contain catalytic Cys111Ser variants (PDB IDs: 6WRH [74], 6XG3 [74], 7CJD [39], 7D47, 7D6H [84], 7D7K [85], 7YBG, and 8FWN). Additionally, six structures feature Cys111Ser variants of enzyme–substrate complexes (PDB IDs: 6YVA [86], 6XAA [58], 6XA9 [58], 7UV5 [80], 7RBS [80], and 7RBR [80]), and one structure includes a bound Ub variant bound to the enzyme, inhibiting its activity (PDB ID: 8CX9 [87]). The remaining structures comprise the enzyme–inhibitor complexes.

**Figure 5:**
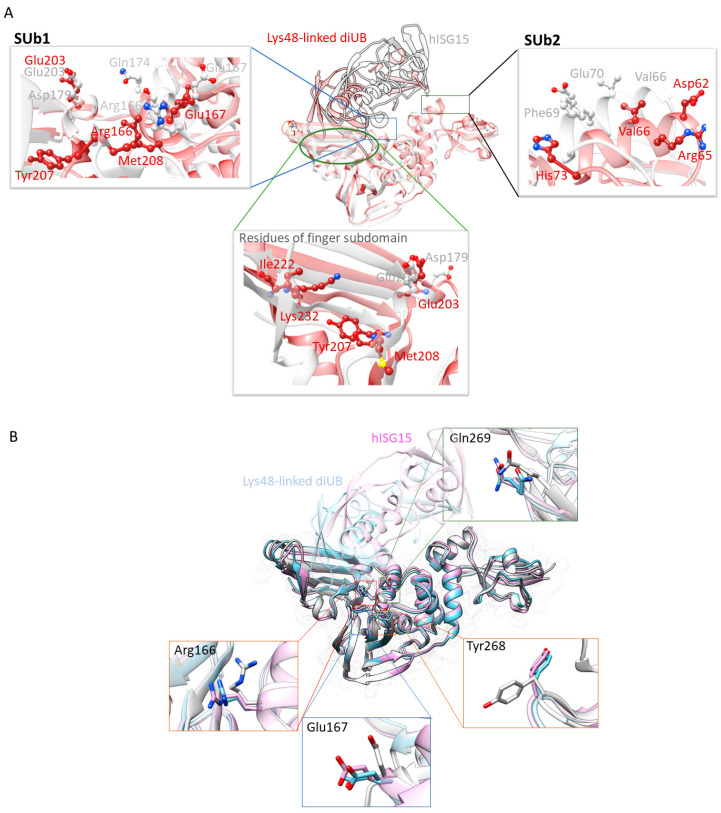
Superimposition of 3D structures of SARS CoV-2 PLpro complexed with hISG15 (PDB ID: 7RBS, white) and Lys48-linked diUb (PDB ID: 7UV5, brown) (**A**). The left and right panels show enlarged views of the protein residues involved in the SUb1 and SUb2 substrate binding pockets, that interact with the hISG15 (white) and Lys48-linked diUb (brown) while the bottom panel highlights residues in the finger subdomain interacting with the same substrates. More residues are observed interacting with Ub compared to ISG15. The superimposition of the 3D structures of apo SARS-CoV-2 PLpro (PDB ID: 8FWN, light gray), hISG15-bound PLpro (PDB ID: 7RBS, baby pink), and diUB-bound PLpro (PDB ID: 7UV5, light blue) (**B**). The residues shown in the panels are all SARS-CoV-2 PLpro residues: light gray represents residues in the apo-protein, and baby pink and light blue indicate residue conformations when the protein is bound to hISG15 and Lys48-linked diUb, respectively. The enlarged views showcase the residues undergoing conformational changes upon substrate binding.

A structural analysis of SARS CoV-2 PLpro, complexed with different substrates, provides crucial insight into the molecular interactions involved in substrate recognition. While the protein’s 3D conformation remains largely unchanged, substrate conformations vary, suggesting that each substrate adopts a unique binding mode. To further investigate these subtle conformational changes, we superimposed the apoprotein structure (PDB ID: 8FWN) with PLpro bound to Lys48-linked diUb (PDB ID: 7UV5) and human ISG15 (PDB ID: 7RBS). Our analysis revealed that more residues from the enzyme’s finger subdomain were involved in the substrate binding envelope (within 3.5 Å of the substrate protein) for Lys48-linked diUb than human ISG15, highlighting the more extensive interactions between Lys48-linked diUb and the finger subdomain (Figure 5A). Additionally, residues Arg166, Glu167, Tyr268, and Gln269 displayed conformational changes upon substrate binding (Figure 5B).

Further exploration of the conformational transitions and residue flexibility in SARS-CoV-2 PLpro, especially during substrate binding, would require in silico methods such as molecular dynamics or metadynamics, which are beyond the scope of this review.

A closer examination of the substrate’s C-terminal extension, which contains the ‘LXGG’ peptide motif, revealed hydrogen bonding interactions with Gly163, Asp164, Gly271, and Tyr273 at the S3/S4 binding sites (Figure 1). Additional interactions were observed at the SUb1 and SUb2 sites (Figure 5A). Understanding these key interactions between the enzyme and its substrate provides insight into the mechanisms by which inhibitors may block PLpro’s function.

SARS-CoV-2 PLpro inhibitors, or cysteine protease inhibitors in general, can be classified into two categories: covalent and non-covalent inhibitors. Covalent inhibitors irreversibly inhibit enzyme function by forming a thioether bond with the catalytic Cys111 residue [79]. Non-covalent inhibitors, in contrast, bind PLpro through non-covalent interactions such as hydrogen bonds, hydrophobic forces, and van der Waals interactions.

There are four 3D structures of SARS-CoV-2 PLpro complexed with covalent ligands (PDB IDs: 6WX4 [79], 6WUU [79], 8IHO [88], and 8UVM [89]), while 48 structures depict non-covalent interactions with various inhibitors, suggesting that non-covalent ligand interactions have been more extensively studied. These ligands bind at substrate recognition sites, including S4, SUb2, SUb1, and the Zn(II) finger (ZF) region. The binding sites, ligands, and PDB IDs for these 3D structures are listed in Table 1.

Using Chimera and visual inspection, we identified the residues involved in binding to ligands, which are at a distance of 3.5 Å or less from the ligands. When multiple enzyme–inhibitor complexes were available for the same ligand, the highest-resolution structure was used to identify interacting residues. Below, we provide a detailed overview of the binding interaction network at these binding sites and highlight inhibitor selectivity.

## 5. Ligands Binding Substrate Recognition Sites S3 and S4

Given the lack of well-defined substrate binding sites S1 and S2, inhibitors have been developed to target the more accessible substrate recognition sites S3 and S4. These inhibitors often span multiple substrate recognition sites, likely due to the structural configuration of the enzyme’s active site. The proximity of these sites (Figure 3) facilitates the binding of inhibitors to both S3 and S4, which are located approximately 8–10 Å from the catalytic triad. A notable structural feature of this ligand binding region is the flexible BL2 loop (residues 267–272) which acts as a lid over the region [34,56]. This loop plays a pivotal role in regulating substrate access to the active site [96]. Though poorly conserved among SARS-CoV PLpro enzymes, the flexibility and variability of the BL2 loop suggest it can accommodate structurally diverse inhibitors. In the unliganded state, the loop generally adopts an open conformation but can transition between open and closed states depending on the ligand size. This flexibility offers opportunities for designing selective inhibitors by adjusting ligand size. In the closed conformation, the BL2 loop flips over, blocking substrate access and forming an extended hydrophobic binding groove adjacent to the loop, referred to as the BL2 groove. This groove engages in interactions that stabilize the inhibitor within the binding site [91].

**Figure 6:**
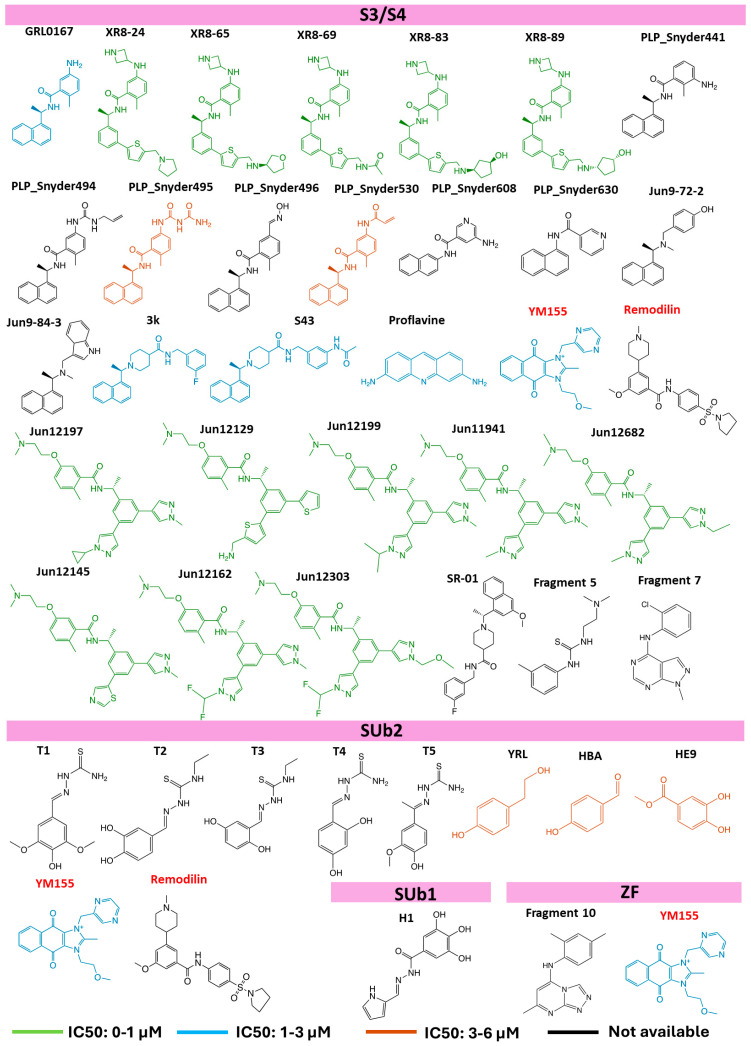
Chemical structures of ligands and their binding sites. The names of the ligands that bind to multiple sites are highlighted in red. The structures are color-coded based on their binding affinities: strong (0–1 μM) in light green, moderate (1–3 μM) in sky blue, and weak (3–6 μM) in brown. The ligands for which affinity data are unavailable are shown in black.

There are 36 3D structures with non-covalent ligands bound to the S3/S4 substrate recognition sites (Table 1). However, these 36 structures correspond to only 29 distinct ligands that bind to the S3/S4 sites and have molecular weights ranging from 200 to 550 Da, as some are represented in multiple crystal structures (Figure 6). This suggests that the S3/S4 sites are key targets for drug discovery. The primary inhibitory mechanism of ligands binding to these sites is the prevention of substrate entry into the active site. The binding pocket is predominantly hydrophobic, with residues Lys157, Leu162, Gly163, Asp164, Glu167, Met208, Pro248, Tyr264, Gly266, Asn267, Tyr268, Gln269, Tyr273, and Thr301 interacting with ligands within 3.5 Å (Table 2). The abundance of non-polar and aromatic residues suggests that π-π or CH/NH-π interactions are the main forces driving ligand binding.

**Figure 7:**
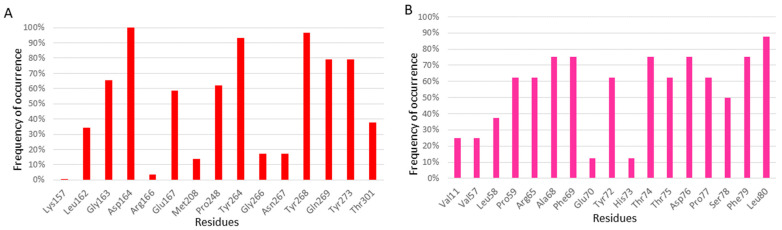
Frequency of amino acid residues involved in ligand binding for pockets S3/S4 (**A**) and SUb2 (**B**).

A residue frequency analysis revealed key binding residues. Asp164 interacts with all the ligands in these structures (Figure 7A), indicating its key role in ligand binding. Mutagenesis studies showed that mutating Asp164 impairs enzymatic activity [97,98], supporting this frequency analysis finding. Tyr264, Tyr268, Gln269, and Tyr273 interact with over 80% of the ligands (Figure 7A), indicating their importance in ligand binding. Tyr268 and Gln269, located in the BL2 loop, play a key role in the loop’s transition between open to closed states. For example, when binding to an aromatic ligand, Tyr268 flips to establish a T-shaped π-π interaction, sealing off the active site from substrate entry [39]. The importance of Tyr268 is further illustrated by the ineffectiveness of the naphthalene-based inhibitor GRL0617 against MERS-CoV, likely due to a threonine replacing tyrosine at position 268 [86]. Tyr264 and Tyr 268, and Pro247 have also been reported to significantly impact ligand binding [98].

The rich presence of aromatic residues in the S3/S4 site offers a unique potential for binding two π-stacked aromatic ligand molecules, as demonstrated with proflavine. Two π-stacked proflavine molecules occupy the S3/S4 site in the 3D structure with PDB ID: 7NT4 [93]. Tyrosine and proline residues interact with these aromatic molecules through π-π stacking, facilitating dual binding modes, both essential for enzyme inhibition.

GRL0617, a well-studied non-covalent PLpro inhibitor, serves as a positive control in inhibitor screening [57]. Comprising naphthalene and benzamide fragments, GRL0617’s naphthalene occupies the S4 site while benzamide binds in the S3 site. Four 3D structures of SARS-CoV-2 PLpro complexed with GRL0617 have been deposited in the PDB (PDB IDs: 7CJM [57], 7CMD [39], 7JIR [74], and 7JRN [90]), including two Cys111Ser variants. Its well-characterized interaction with PLpro makes GRL0617 a valuable reference for evaluating new inhibitors. Several GRL0617 derivatives, such as the XR8, Snyder, and Jun-series ligands, have been synthesized to enhance potency and binding affinity [74,91].

**Figure 8:**
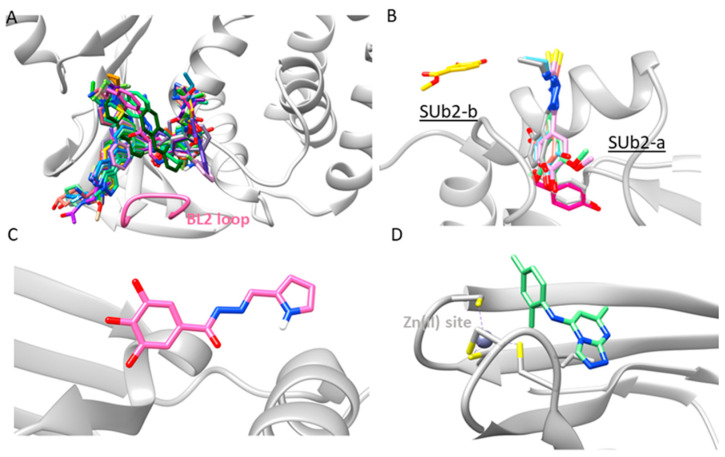
Binding modes of the SARS CoV-2 PLpro inhibitors in the substrate recognition sites S3/S4 (**A**), SUb2 (**B**), SUb1 (**C**), and ZF region (**D**). The inhibitors are represented in a stick model.

Superimposing the inhibitor-bound 3D structures (Figure 8A) revealed that GRL0617 derivatives exhibit similar binding orientations of the naphthalene and benzamide fragments within the binding pocket. Modifications in these derivatives could enhance interactions with the BL2 groove or the S3 site. Extensions into the BL2 groove are solvent-exposed, while those into the S3 site engage deeper into the enzyme’s core, suggesting a potential strategy for designing more effective inhibitors by balancing solvent exposure with deep-pocket engagement.

In addition to GRL0617, two small fragments, fragment 5 and fragment 7, exhibit inhibitory activity and could serve as the starting points for designing new inhibitors [94]. Recently, the deposited 3D structures of Jun-series ligands [89], including Jun12197, Jun12129, Jun12199, Jun11941, Jun12682, Jun12162, and, Jun12145, show interference with Val70 of Ub or Asn151 of ISG15. This interference suggests that inhibitors targeting the S3 and S4 sites may offer the multi-functional inhibition of PLpro, providing new avenues for developing effective therapeutics by exploiting the enzyme’s conformational flexibility.

## 6. Ligands Binding Substrate Recognition Site SUb2

The SUb2 substrate recognition site for Ub/ISG15 is an allosteric site located about 30 Å from the catalytic center of the enzyme. This site is situated near the α2 and α3 helices within the thumb domain, forming a deep binding pocket [95]. Sharing 90% similarity and 83% identity in the sequences of SARS-CoV PLpro and SARS-CoV-2 PLpro [79], Sub2 interacts with the N-terminal domains of Ub/ISG15 substrates [80]. While less explored than the S3/S4 sites, the inhibitors targeting the SUb2 site may alter its flexibility, thereby disrupting the binding of ISG15 or Ub to PLpro.

We identified eight SARS-CoV-2 PLpro 3D structures with inhibitors bound to the SUb2 site (Table 1, Figure 6). Upon overlaying these structures (Figure 8B), we observed two distinct ligand binding locations within the SUb2 site. Seven ligands, with molecular weights ranging from 122 to 255 Da, are deeply embedded within the enzyme [83,95], while one ligand, with a molecular weight of 168 Da, occupies the enzyme’s surface [83]. We designate these sites as SUb2-a and SUb2-b.

Our analysis identified the key residues within 3.5 Å of these ligands. The SUb2-a site is surrounded by 11 residues, seven of which are hydrophobic, while the rest are polar or polar charged. These residues include Val11, Tyr56, Val57, Pro59, Arg65, Ala68, Phe69, Tyr72, Thr75, Phe79, and Leu80 (Table 2). Notably, Leu80 exhibited the highest interaction frequency (>85%) across all the ligands (Figure 7B). Others frequently interacting (>70%) include Ala68, Phe69, Thr74, Asp76, and Phe79. The primary interactions are hydrogen bonds, hydrophobic contacts, and van-der Waals forces. Key residues such as Arg65, Phe69, Glu70, and Thr75, which also interact with ISG15/Lys48-linked diUb, play a crucial role in ligand binding. Mutagenesis studies further emphasize the indispensable role of Phe69 in substrate binding [40,86,99], highlighting the SUb2 region’s critical involvement in deubiquitination over ISG15-ylation.

The inhibitors binding to SUb2 are categorized into two main classes: thiosemicarbazones and polyphenols. While these inhibitors generally exhibit low inhibitory activity or binding affinity, they are strategically positioned to disrupt protein–protein interactions, making them promising candidates for optimization [83,95]. Thiosemicarbazones (T1–T5) exhibit a similar binding mode, as shown by their structure overlap. Polyphenols HBA and YRL also share consistent binding patterns, though their overlap with thiosemicarbazones is partial. Thiosemicarbazones bind near SUb2-a and can cause steric hindrance, which impacts enzyme–substrate interactions and expands the binding site. Ewert and coworkers proposed extending thiosemicarbazones with the phenolic fragments of HBA and YRL to enhance hydrophobic interactions [95], leading to tighter binding and the potential for developing more potent inhibitors.

The SUb2-b site, located on the surface of PLpro and adjacent to the SUb2-a, includes residues Phe69, Glu70, and His73—known contributors to substrate binding. The inhibitory mechanism at SUb2-b likely mirrors that of SUb2-a, with ligands disrupting protein–protein interactions or inducing conformational changes that impede enzyme function. As a solvent-exposed binding site, SUb2-b may favor ligands with hydrophilic groups. Only one structure, a polyphenol (HE9), was found to bind at SUb2-b. Superimposing this structure with Lys-48 linked diUB/ISG15 suggested that Glu70 reorients upon ligand binding to interact with the inhibitor. Although YRL, HBA, and HE9 have shown inhibition in deISGylation assays [83], their effects on ubiquitination remain unclear, warranting further investigation to fully understand their inhibitory mechanisms and selectivity.

## 7. Ligands Binding Substrate Recognition Site SUb1

The allosteric SUb1 binding site is a polar groove located approximately 20 Å away from the catalytic triad [83]. To date, only one 3D structure of SARS-CoV-2 PLpro has been reported with a ligand, hydrazone (H1), bound to the SUb1 region (Figure 6 and Figure 8C), suggesting that this is one of the least explored inhibitor binding sites. The binding cavity of H1 consists of six polar and two non-polar residues, including Arg166, Ser170, Tyr171, Gln174, Glu203, Met206, Tyr207, and Met208 (Table 2). Hydrogen bonding plays a key role in ligand interaction with PLpro.

When the 3D structure is superimposed with the enzyme–substrate complex (PDB ID: 7RBS and 7UV5), the ligand partially overlaps with the N-terminal substrate binding region, resulting in steric hindrance and destabilizing substrate binding. Additionally, changes in the orientations of key residues Arg166 and Glu203, involved in substrate binding, are observed upon H1 binding. Arg166, in particular, appears to play a significant role in substrate recognition, maintaining hydrogen bonding interactions with the N-terminal residues of the substrate Ub/ISG15, while also forming electrostatic interactions with Asp164, a key residue of SARS-CoV-2 PLpro [90,97]. Although its exact function is not fully understood, Arg166 may contribute to substrate stabilization and potentially influence enzymatic activity.

## 8. Ligands Binding Zn(II) Finger Region

The Zn(II) finger region, located approximately 40 Å away from the catalytic site, is another poorly explored site for potential allosteric inhibition. Its role in proteolytic activity has been demonstrated through mutagenesis studies targeting the Zn(II)-coordinating cysteine residues [100]. A fragment-based screening identified a triazolo pyrimidine-based fragment 10 (Figure 6 and Figure 8D) bound near the Zn(II) binding site [94]. While this fragment showed no inhibitory activity against PLpro [94], its binding to PLpro revealed a new druggable site. The binding cavity, formed by residues Val187, Cys192, Pro223, Cys224, and Thr225, within 3.5 Å from the fragment (Table 2), appears to exhibit structural flexibility.

Superimposing this 3D structure with Lys48-linked diUb (PDB ID: 7UV5) highlights that this region, particularly compared to other inhibitor binding sites, exhibits the highest degree of flexibility. Aside from slight positional shifts due to its inherent flexibility, no significant conformational changes were observed, which may explain the fragment’s lack of inhibitory activity. Nevertheless, the Zn(II) binding region remains crucial for maintaining PLpro’s structural stability [58,100]. Disrupting the tetrahedral coordination of this site through targeted modifications could lead to enzyme destabilization and inhibition.

## 9. Ligands Binding Multiple Sites

**Figure 9:**
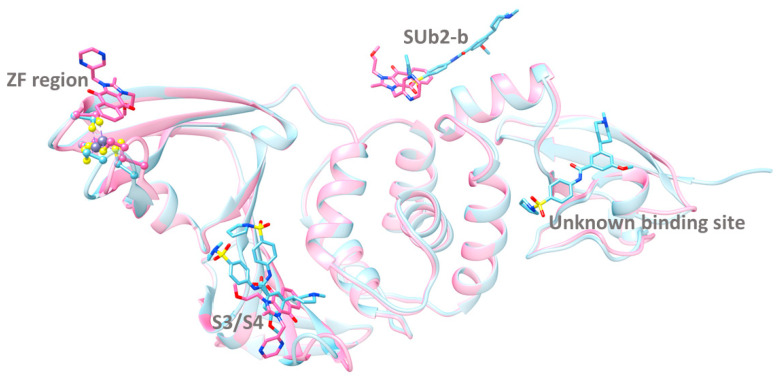
Superimposition of the 3D structures of SARS CoV-2 PLpro bound with YM155 (pink) and remodilin (blue). The inhibitors are represented in a stick model.

In addition to ligands targeting specific sites like S4, SUb2, SUb1, and ZF, some inhibitors have been found to bind multiple sites within PLpro. Notable examples include the imidazolium-based compound YM155 and the benzamide-based compound remodilin (Figure 6). YM155, with a molecular weight of 363 Da, binds to three distinct sites in chain A of the enzyme: S3/S4, SUb2-b, and the Zn(II) finger region (Figure 9). In chain B, however, it binds only to the S3/S4 and SUb2-b sites [85]. The planar imidazole-based core skeleton of YM155, enriched with electron-donating nitrogen atoms, plays a crucial role in enabling interactions within the three binding cavities.

Remodilin, a larger ligand with a molecular weight of 457 Da, binds to the S3/S4, SUb2-b, and an unidentified site located at the interface between the Ubl and thumb domains in chain A (Figure 9). In chain B, remodilin binds solely to the S3/S4 site, while in chain C, it binds to both the S3/S4 and SUb2-b sites. The unidentified binding site may be an artifact, but no further details have been published. Interestingly, in all three chains, remodilin adopts two different conformations in the S3/S4 site, indicating its structural flexibility.

The structural versatility of these compounds underpins their ability to engage with multiple binding environments. Both compounds share a combination of aromatic scaffolds and polar functional groups, enabling them to form hydrophobic and hydrophilic interactions within binding sites. These multi-site binders may employ unique inhibitory mechanisms by interacting with various regions of the protein, enhancing both binding affinity and inhibitory potency. The ability to target multiple binding sites simultaneously could produce synergistic effects, resulting in stronger inhibition than binding at individual sites alone.

## 10. Concluding Remarks and Perspectives

PLpro is a promising drug target in the fight against COVID-19 due to its dual roles in cleaving viral polyproteins and modulating host deubiquitination and deISGylation activities [80,101]. However, the current PLpro-based inhibitors have not yet demonstrated the pharmacokinetics and potency necessary for clinical trials. Structural and mechanistic insights from X-ray crystallography can aid computer-aided drug discovery by offering detailed information on the enzyme’s active sites, binding pockets, and interaction networks, facilitating the design of more potent and selective inhibitors.

The structural analysis revealed that the distinct structural and chemical properties of PLpro’s binding sites play a key role in accommodating ligands with varying structural features. These insights highlight the importance of designing ligands tailored to exploit the unique characteristics of each binding site. While the S3/S4 site has been a focal point in drug discovery, other substrate recognition sites, including SUb1, SUb2, and the Zn(II) finger region, remain underexplored, partly due to the limited availability of crystal structures. Expanding structural knowledge through advanced techniques like crystallography, cryo-EM, and nuclear magnetic resonance will be crucial in unlocking the therapeutic potential of these underutilized substrate recognition sites.

Inhibitors like YM155 and Remodilin, which engage multiple sites within PLpro, suggest a promising strategy for enhancing inhibition and potentially reducing resistance. However, additional biochemical and kinetic studies are needed to complement the structural data. Leveraging computational methods such as molecular dynamics simulations and virtual screening will provide deeper insight into the dynamics of multi-site binding, paving the way for the development of more robust inhibitors.

PLpro holds significant potential for future drug discovery, particularly in the fight against COVID-19. Its diverse array of ligand binding pockets offers opportunities to design structurally diverse ligands that can exploit both hydrophobic and polar interactions within the binding sites. With continued advancements in structural biology, PLpro is positioned to emerge as a pivotal therapeutic target, opening new avenues for the development of highly selective and effective inhibitors.

## Figures and Tables

**Table 1:** Classification of the X-ray crystallographic structures based on the ligand binding site.

Binding Site		PDB ID	Resolution (Å)	Inhibitor	Reference
S3/S4		7CJM	3.20	GRL0617	[57]
		7CMD	2.59		[39]
		7JRN	2.48		[90]
		7JIR	2.09		[74]
		7LBS	2.80	XR8-24	[91]
		7LOS	2.90	XR8-65	
		7LLZ	2.90	XR8-69	
		7LLF	2.30	XR8-83	
		7LBR	2.20	XR8-89	
		7JN2	1.93	PLP_Snyder441	
		7KRX	2.72		
		7KOJ	2.02	PLP_Snyder494	
		7JIT	1.95	PLP_Snyder495	[74]
		7KOK	2.00	PLP_Snyder496	
		7KOL	2.58		
		7JIW	2.30	PLP_Snyder530	[74]
		7SGU	1.79	PLP_Snyder608	
		7SGV	2.00	PLP_Snyder630	
		7SGW	1.95		
		7SDR	2.72	Jun9-72-2	
		7RZC	2.04	Jun9-84-3	
		7SQE	2.00		
		7TZJ	2.66	3k	[92]
		7E35	2.40	S43	[84]
		7NT4	2.68	Proflavine	[93]
		8UUV	3.01	Jun12197	[89]
		8UUY	3.05	Jun12129	
		8UUH	2.80	Jun12199	
		8UUF	2.84	Jun11941	
		8UOB	2.52	Jun12682	
		8UUU	3.01	Jun12162	
		8UUW	3.20	Jun12145	
		8UUG	2.74	Jun12303	
		8JUX	3.20	SR-01	
		9BRV	2.60	Fragment 5	[94]
		9BRW	2.50	Fragment 7	
SUb2	SUb2-a	7QCH	1.88	T1	[95]
		7QCI	1.76	T2	
		7QCK	1.92	T3	
		7QCJ	1.84	T4	
		7QCM	1.77	T5	
		7OFS	1.90	YRL	[83]
		7OFT	1.95	HBA	
	SUb2-b	7OFU	1.72	HE9	
SUb1		7QCG	1.75	H1	[95]
ZF		9BRX	1.80	Fragment 10	[94]
Multi-site binding ligands					
S3/S4, SUb2, ZF		7D7L	2.11	YM155	[85]
S3/S4, SUb2, Unidentified site		8G62	2.17	Remodilin	

**Table 2:** Residues involved in binding interactions with ligands.

**S3/S4**		
**PDB ID**	**Ligand**	**Binding Residues**
7JRN	GRL0617	Asp164, Pro248, Tyr264, Tyr268, Gln269, Tyr273, Thr301
7LBS	XR8	Leu162, Glu167, Asp164, Pro248, Tyr264, Tyr268, Gln269, Tyr273, Thr301
7LOS	XR8-65	Asp164, Gly266, Tyr268, Gln269, Thr301
7LLZ	XR8-69	Gly163, Asp164, Glu167, Tyr264, Tyr268, Gln269, Thr301
7LLF	XR8-83	Gly163, Asp164, Pro248, Tyr264, Gln269
7LBR	XR8-89	Leu162, Gly163, Glu167, Asp164, Pro248, Tyr264, Tyr268, Gln269, Tyr273, Thr301
7JN2	PLP_Snyder441	Gly163, Glu167, Asp164, Pro248, Tyr264, Tyr268, Gln269, Tyr273
7KOJ	PLP_Snyder494	Gly163, Asp164, Glu167, Pro248, Tyr264, Tyr268, Gln269, Thr301
7JIT	PLP_Snyder495	Gly163, Glu167, Asp164, Pro248, Tyr264, Tyr268, Gln269, Thr301
7KOK	PLP_Snyder496	Gly163, Asp164, Glu167, Pro248, Tyr264, Tyr268, Gln269, Thr301
7JIW	PLP_Snyder530	Gly163, Glu167, Asp164, Pro248, Tyr264, Gln269, Tyr273
7SGU	PLP_Snyder608	Asp164, Tyr264, Gln269, Tyr273
7SGW	PLP_Snyder630	Asp164, Tyr264, Tyr268, Gln269, Tyr273
7SDR	Jun9-72-2	Lys157, Asp164, Glu167, Tyr264, Tyr268, Tyr273
7SQE	Jun9-84-3	Asp164, Glu167, Pro248, Tyr268, Tyr273
7TZJ	3k	Leu162, Gly163, Asp164, Met208, Pro248, Tyr264, Asn267, Tyr268, Gln269, Tyr273, Thr301
7E35	S43	Leu162, Asp164, Pro248, Tyr264, Tyr268, Gln269, Tyr273, Thr301
7NT4	Proflavine	Gly163, Asp164, Pro248, Tyr264, Tyr268, Tyr273
8JUX	SR-01	Asp164, Pro248, Tyr264, Asn267, Tyr268, Tyr273, Thr301
8UUV	Jun12197	Gly163, Asp164, Glu167, Pro248, Tyr264, Gly266, Tyr268, Gln269, Tyr273
8UUY	Jun12129	Leu162, Gly163, Asp164, Tyr264, Gly266, Tyr268, Gln269, Tyr273
8UUH	Jun12199	Leu162, Gly163, Asp164, Arg166, Glu167, Pro248, Tyr264, Tyr268, Gln269, Tyr273
8UUF	Jun11941	Gly163, Asp164, Glu167, Pro248, Tyr264, Gly266, Tyr268, Gln269, Tyr273
8UOB	Jun12682	Leu162, Gly163, Asp164, Glu167, Met208, Tyr264, Tyr268, Gln269, Tyr273,
8UUU	Jun12162	Gly163, Asp164, Glu167, Tyr264, Tyr268, Gln269, Tyr273
8UUW	Jun12145	Leu162, Gly163, Asp164, Glu167, Met208, Tyr264, Gly266, Asn267, Tyr268, Gln269, Tyr273
8UUG	Jun12303	Leu162, Gly163, Asp164, Glu167, Met208, Pro248, Tyr264, Asn267, Tyr268, Gln269, Tyr273
9BRV	Fragment 5	Gly163, Asp164, Tyr264, Tyr273
9BRW	Fragment 7	Leu162, Asp164, Tyr264, Asn267, Tyr268, Tyr273
**SUb2**		
**PDB ID**	**Ligand**	**Binding residues**
7QCH	T1	Leu58, Pro59, Arg65, Ala68, Phe69, Tyr72, Thr74, Thr75, Asp76, Pro77, Ser78, Phe79, Leu80
7QCI	T2	Leu58, Pro59, Arg65, Ala68, Phe69, Thr74, Thr75, Asp76, Pro77, Phe79, Leu80
7QCJ	T4	Pro59, Arg65, Ala68, Phe69, Tyr72, Thr74, Thr75, Asp76, Pro77, Ser78, Phe79, Leu80
7QCK	T3	Pro59, Arg65, Ala68, Phe69, Tyr72, Thr74, Thr75, Asp76, Pro77, Ser78, Phe79, Leu80
7QCM	T5	Leu58, Pro59, Arg65, Ala68, Phe69, Thr74, Thr75, Asp76, Pro77, Ser78, Phe79, Leu80
7OFS	YRL	Val11, Val57, Ala68, Tyr72, Thr74, Asp76, Leu80
7OFT	HBA	Val11, Val57, Tyr72, Phe79, Leu80
7OFU	HE9	Phe69, Glu70, His73
**SUb1**		
**PDB ID**	**Ligand**	**Binding residues**
7QCG	H1	Arg166, Ser170, Tyr171, Gln174, Glu203, Met206, Tyr207, Met208
**ZF**		
**PDB ID**	**Ligand**	**Binding residues**
9BRX	Fragment 10	Val187, Cys192, Pro223, Cys224, Thr225

## Data Availability

No new data were created or analyzed in this study. Data sharing is not applicable.
